# Vimentin protects differentiating stem cells from stress

**DOI:** 10.1038/s41598-020-76076-4

**Published:** 2020-11-11

**Authors:** Sundararaghavan Pattabiraman, Gajendra Kumar Azad, Triana Amen, Shlomi Brielle, Jung Eun Park, Siu Kwan Sze, Eran Meshorer, Daniel Kaganovich

**Affiliations:** 1 Department of Experimental Neurodegeneration, University Medical Center Göttingen, Waldweg 33, 37073 Göttingen, Germany; 2 Base Pharmaceuticals, Boston, MA 02129 USA; 3 Present Address: Department of Genetics, The Institute of Life Sciences, The Hebrew University of Jerusalem, Edmond J. Safra Campus, 91904 Jerusalem, Israel; 4 The Edmond and Lily Safra Center for Brain Research (ELSC), The Hebrew University of Jerusalem, Edmond J. Safra Campus, 91904 Jerusalem, Israel; 5 School of Biological Sciences, Nanyang Technological University, 60 Nanyang Drive, Singapore, 637551 Singapore; 6 Department of Zoology, Patna University, Patna, 800005 India

**Keywords:** Cytoskeleton, Intermediate filaments

## Abstract

Vimentin is one of the first cytoplasmic intermediate filaments to be expressed in mammalian cells during embryogenesis, but its role in cellular fitness has long been a mystery. Vimentin is acknowledged to play a role in cell stiffness, cell motility, and cytoplasmic organization, yet it is widely considered to be dispensable for cellular function and organismal development. Here, we show that Vimentin plays a role in cellular stress response in differentiating cells, by recruiting aggregates, stress granules, and RNA-binding proteins, directing their elimination and asymmetric partitioning. In the absence of Vimentin, pluripotent embryonic stem cells fail to differentiate properly, with a pronounced deficiency in neuronal differentiation. Our results uncover a novel function for Vimentin, with important implications for development, tissue homeostasis, and in particular, stress response.

## Introduction

Vimentin is a type III intermediate filament (IF), and is one of the first cytoplasmic filaments to be expressed in a developing organism^1^, as early as day 8 of embryogenesis, after Keratin expression^2^. Embryonic Stem Cells (ESCs) have low levels of Vimentin, which is turned on early in differentiation, and is later replaced by tissue-specific intermediate filaments in most cell types^1,2^. It is composed of coiled-coil alpha-helices with flexible head and tail regions, which vary between different type III IFs such as Glial Fibrillary Acidic Protein (GFAP), Neurofilaments, Desmin, and Peripherin; and two coiled-coil rod domains which are conserved between type III IFs^3,4^. Vimentin is normally localized in ultrastructurally diverse filaments spanning from the nuclear periphery to the cell membrane and associates with the actin and tubulin cytoskeleton^5^, with organelles, including mitochondria and lipid droplets, and with chaperones like αβ-crystallin^5–8^. It has also been shown to interact with several signaling proteins, including 14-3-3 proteins, Extracellular signal-regulated kinase (ERK), and Rho-associated protein kinase (RhoK)^9–11^. The cellular functions of Vimentin are not completely established, although it was shown to contribute to cell stiffness, cell motility, actin positioning, and organelle trafficking^6,12,13^.

Despite the early and ubiquitous expression of Vimentin, its physiological role has been unclear. The *Vimentin*-/- knockout mouse displays few reported abnormalities, aside from poor wound healing, a smaller carotid artery, and intestinal defects^14–16^. One study recently showed that murine embryonic stem cells (mESCs) from Vimentin-/- mice have slower Embryoid Body (EB) growth relative to wild-type^17^. It has also been observed that Mouse Embryonic Fibroblasts (MEFs) lacking Vimentin are difficult to immortalize and delay entry into senescence^18^. Indeed, Vimentin is implicated in tumorigenesis, since it is highly upregulated during the epithelial-mesenchymal transition, and there are studies showing that Vimentin is needed for metastasis^19–21^. It also seems to facilitate certain viral infections^22^. Recent work has additionally shown that Vimentin modulates inflammation in macrophages during atherogenesis and regulates NOTCH signaling during angiogenesis^23,24^. All this suggests that Vimentin confers a protective or pro-survival function on cells^9,25^ and may specifically be involved in heat-stress tolerance^9^.

In recent work, we showed that Vimentin is partitioned asymmetrically in dividing immortal cell lines^26^. A subsequent study reported the asymmetric partitioning of Vimentin together with ubiquitinated proteins in developing neuronal progenitor cells (NPCs) away from the differentiating neuron^27^. Together these and other observations led us to propose a role for Vimentin in replicative rejuvenation—the process of asymmetrically partitioning aggregated proteins and other damaged components during mitosis, so as to produce a pristine lineage^27–32^. In support of this, a very recent study identified a Vimentin mutation in an individual presenting with a premature aging pathology^33^. However, an unequivocal physiological requirement for Vimentin in stress response or rejuvenation is yet to be demonstrated.

We set out to systematically examine the requirement of Vimentin for mouse ESC neuronal differentiation and stress tolerance. Using CRISPR/Cas9 knock-out mESC lines, we show that Vimentin is critical for stress tolerance in differentiating, but not in pluripotent, stem cells. Examining the Vimentin interactome during differentiation and stress revealed that Vimentin protects cells by associating with aggregates and Stress Granules, and directing their asymmetric partitioning during mitosis. High temporal and spatial resolution imaging in live cells showed that Vimentin does not simply encage aggregates, as has been suggested previously^26,34,35^. Instead, Vimentin colocalizes with aggregate foci and Stress Granules throughout the cytoplasm, and recruits them to a retracted Vimentin mesh-like structure. Vimentin knock-out cells had dramatically altered gene expression and differentiation profiles. Cells deleted for Vimentin fail to differentiate into neuronal progenitors, and the defect is severely exacerbated during stress. Our data suggest that Vimentin may be dispensable during normal conditions, but is critically important when cells are exposed to protein folding stress.

## Results

### Vimentin protects differentiating stem cells from aggregate toxicity

**Figure 1:**
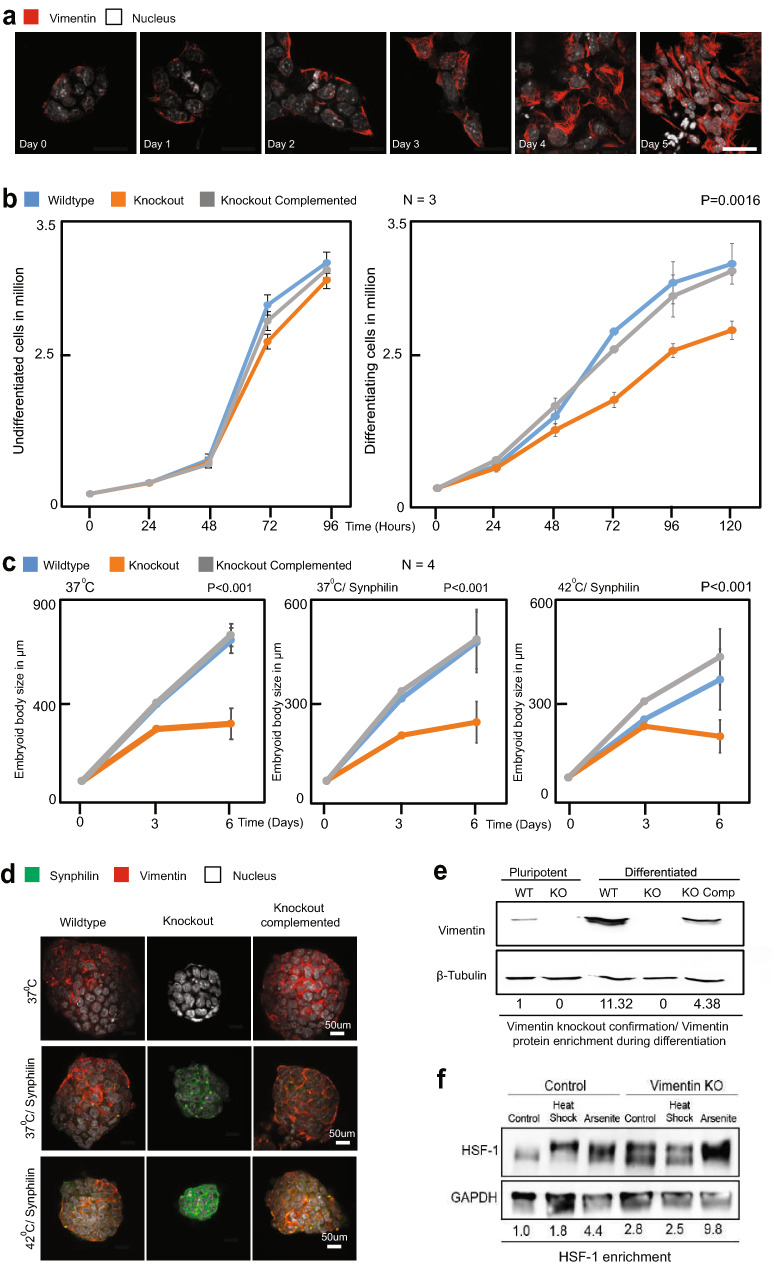
Vimentin Knockout cells are defective in growth and differentiation. (**a**) mESCs differentiated by the addition of retinoic acid (1 µg/ml) and fixed every 24 h. Immuno-fluorescence for vimentin (red) and nucleus (white) is shown at each time point. Increase in the expression of vimentin was observed at each time point. Images were acquired and processed using NIS elements software (version 3.2) (**b**) Growth curve comparison between (1) Wildtype, (2) Knockout and (3) Knockout complemented with full-length vimentin in cells maintained in stem cell (2i) media and retinoic acid (differentiation) media. When the cells are maintained in 2i media, no significant change in the proliferation was observed. During differentiation, the wildtype and knockout complemented cells have higher proliferating potential than the knockout cells. The error bars represent standard deviation, statistics were performed by two tailed t-test (*P* = 0.001).(**c**) Pluripotent stem cells were seeded on to non-adherent plates without the pluripotency factors leading to the formation of embryoid bodies (EBs). Sizes of the EBs were compared between (1) Wildtype, (2) Knockout and (3) Knockout complemented with full-length vimentin. The sizes were measured every 3 days. The wildtype and knockout complemented cells form larger EBs then the vimentin knockout cells. The experiment was repeated 4 times with measuring 25 embryoid bodies. The same experiment was repeated for cells—(1) with the overexpression of the misfolded protein synphilin and (2) overexpression of the misfolded protein synphilin coupled with heat stress. The error bars represent standard deviation, (*P* < 0.001). Statistics were performed using two tailed student t-test. (**d**) Representative images of EBs formed by (1) Wildtype, (2) Knockout and (3) Knockout complemented with full-length vimentin cells. The Vimentin KO was complemented with full length Vimentin-RFP. (**e**) Western blot for Vimentin for both pluripotent and differentiated (with retinoic acid 1 μg/ml), cells were performed. (**f**) Western blot for HSF-1 protein after 4 days of differentiation with retinoic acid (1 μg/ml) in wildtype and vimentin knockout cells in two conditions—(1) No stress (2) Heat stress (42 °C).

Vimentin is expressed at low levels in mESCs (Fig. 1a,e; Supplementary Fig. 1A), but its expression level rises significantly following three days of retinoic acid (RA) differentiation and peaks after four days (Supplementary Fig. 1A). In order to examine the role of Vimentin in ESC differentiation and stress response, we generated a mouse ESC R1 line with a homozygous deletion for Vimentin introduced via CRISPR/Cas9 gene editing (Fig. 1e, Supplementary Fig. 1C–E). Vimentin −/− mESCs exhibited no growth defects (Fig. 1b, left panel), consistent with what was observed in the Vim −/− mouse. However, when we triggered differentiation of the R1 cells by addition of RA, we observed significant growth differences between the Vimentin −/− and WT cells (Fig. 1b, right panel). Allowing mESCs to differentiate into Embryoid Bodies (EBs) revealed a dependency on Vimentin for robust EB growth (Fig. 1c,d), similar to what was reported earlier^17^ in ES cells derived from the Vimentin −/− mouse. Complementing the Vimentin −/− mESCs with virally-integrated RFP-tagged Vimentin completely restored normal growth. Although the growth defect was substantial, the differentiated mESCs and the EBs were nevertheless able to proliferate. We next reasoned that if Vimentin does have a stress-response role, as we hypothesize, culturing EBs under protein folding stress will uncover a greater growth defect.

To test this hypothesis, we expressed aggregation-prone proteins (including mutant SOD1^36^, mutant TDP43, AggDD^37^ , Ubc9ts^31^, and Synphilin-1^38^ from virally integrated vectors in WT and Vimentin−/− mESC cells. We focused on growth assays with Synphilin-1, which exhibited no cytotoxicity in WT cells, and therefore allowed to monitor cell survival and proliferation. Expression of Synphilin did not appreciably exacerbate the growth defect of Vimentin −/− EBs (Compare Fig. 1b,c and Fig. 1d, center panel). However, growing Vimentin −/− EBs expressing Synphilin-1 at mild heat shock temperatures (42 °C) uncovered a notable growth defect over time (Fig. 1c, right panel, d, lower panel). Initially, cells expressing Synphilin-1 grew faster, possibly due to a heat shock response which is cytoprotective. By the 6th day of EB differentiation, however, there was dramatic cell death in the Vimentin −/− Synphilin-1-expressing cells, leading to a significant decrease in average EB size (Fig. 1c,d; Supplementary Fig. 2A,B). Interestingly, cells that were complemented with virally-integrated Vimentin showed greater stress tolerance than WT cells, likely due to earlier and more abundant Vimentin expression, driven by a strong promoter. Indeed, Western Blotting showed 4-times more Vimentin in complemented cells (Fig. 1e, Supplementary Fig. 1B).

In order to assess heat shock response in WT and Vimentin −/− cells, we blotted against the Hsf-1 transcription factor regulator of heat shock response (Fig. 1f). Surprisingly, not only did Vimentin−/− cells show a much higher level of Hsf-1 activation (upper band represents phosphorylated Hsf-1)^39^, but these cells also maintained a baseline heat shock response that was higher than the heat stress-induced Hsf-1 response in WT cells (Fig. 1f, Supplementary Fig. 1F). From the western blot data, it is also evident that Hsf-1 is activated more in vimentin −/− cells compared to WT during stress conditions (Heat and Arsenite), possibly because the absence of Vimentin causes additional protein folding stress and aggregate accumulation^19^. These data decisively establish a protective role for Vimentin during protein folding stress, and indicate that Vimentin−/− cells are constitutively stressed in normal conditions.

### Vimentin interacts directly with protein aggregates, and directs their asymmetric partitioning during mESC differentiation

**Figure 2:**
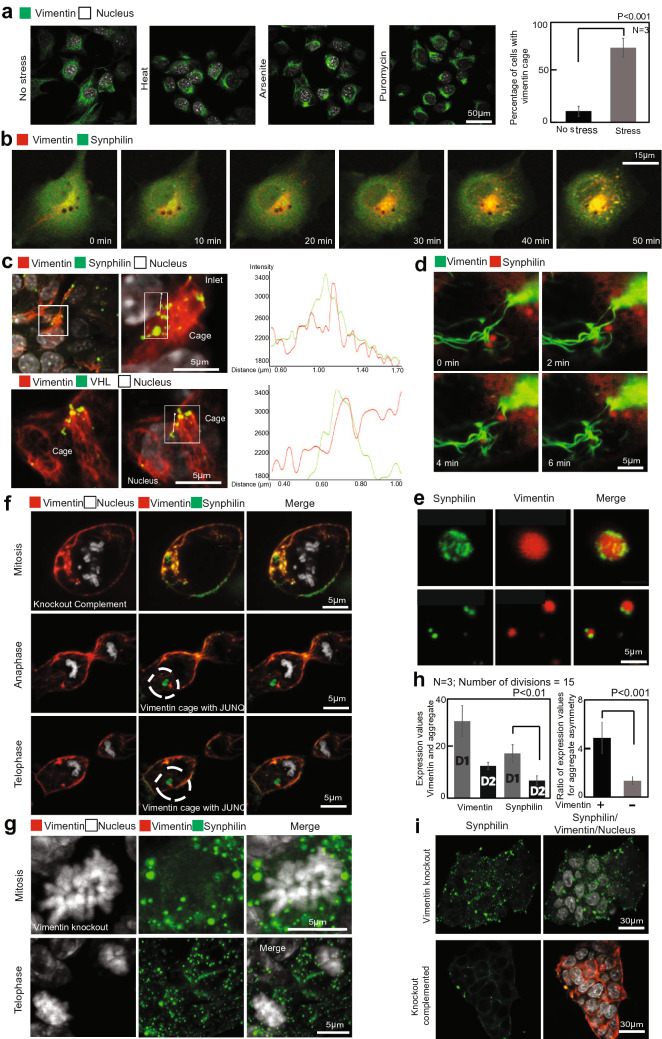
Vimentin protects differentiating stem cells from protein aggregation stress. (**a**) mESCs differentiated with retinoic acid (1 µg/ml) for 4 days. Protein folding stresses (for 2 h) such as (1) Heat (44 °C), (2) Puromycin (4 µg/ml) and (3) Arsenite (200 µM) were induced to the cells and fixed. Immuno-fluorescence for vimentin (green) and nucleus (white) is shown at each time point. During stress, vimentin retracts to the juxtanuclear region. Bottom panels illustrate vimentin (red) collapse in a counterstained cell that is also expressing synphilin (green). Both the vimentin and synphilin foci collapse while the rest of the cell remains intact. Statistics for the cells with vimentin cages compared between (1) no stress (2) stress. The number of cages during all the three stresses were combined and plotted as one value. 100 cells were counted for each repeat. Error bars indicate standard deviation. Statistics were performed using two tailed student t-test. (*P* < 0.001). (**b**) Differentiating mESCs (retinoic acid − 1 µg/ml), overexpressed with Synphilin-GFP and anti-vimentin chromobodies (RFP). Arsenite stress was induced (150 µM/2 h). The vimentin filament collapses along with synphilin aggregates with the cell size being intact. (**c**) Differentiating mESCs (retinoic acid − 1 µg/ml), over-expressed with Synphilin-GFP and VHL-GFP. Confocal images of Vimentin-RFP attaching and forming a cage along with the misfolded protein Synphilin-GFP (Top panel) and VHL-GFP (Bottom panel). Statistics in the right panel show the histogram for the Vimentin—aggregate colocalization. (**d**) Confocal time-lapse of Vimentin (green) recruiting Synphilin (red) to the vimentin cage. (**e**) Vimentin—ULF—GFP (green) interacting with Synphilin—RFP focis (red). (**f**) Confocal imaging of differentiating mESCs with Vimentin (red), Synphilin (green) and Nucleus (white). Top Panels—cells in M-phase with vimentin cage and aggregates. Bottom panels—cells during anaphase and telophase. (**g**) Confocal images of differentiating Vimentin knockout mESCs with Vimentin (red), Synphilin (green) and Nucleus (white). Top Panels—cells in M-phase with aggregates all over the cell. Bottom panels—cells during telophase. In Vimentin knockout cells, the Synphilin aggregates unable to collapse and form a JUNQ is symmetrically inherited during division. (**h**) Statistics for the Vimentin/aggregate asymmetry levels with respect to expression values between the two daughter cells after divisions (*P* < 0.01). Graph for ratio of expression values of the misfolded proteins between the daughter cells after division was plotted between Wildtype and Vimentin Knockout cells (*P* < 0.001). Error bars represent standard deviation. (**i**) Colonies of Vimentin Knockout (top panel) and vimentin complemented (Bottom panel) cells. Knockout cells have higher number of larger Synphilin aggregates (green) after 4 days of differentiation compared to wildtype or knockout complements.

Next, we sought to investigate the mechanism of the protective effect that we observed for Vimentin. Although we have previously observed asymmetric partitioning of Vimentin in cultured cell lines^26^ this has not been demonstrated in dividing mESC cells. Moreover, in previous studies, we only observed an asymmetric partitioning of Vimentin that was retracted into a juxtanuclear “Vimentin cage”. Although Vimentin that is cytoplasmically distributed still partitions in an asymmetric fashion, in most dividing cells a significant sub-cellular pool of Vimentin retracts to the juxtanuclear region prior to metaphase through a known mechanism^40,41^. (Supplementary Movie 1 and 2). Examining the cellular distribution of Vimentin more closely revealed that heat, arsenite, and folding stress (i.e. puromycin), all lead to accelerated retraction of Vimentin into the “cage” or mesh structure, while the rest of the cellular cytoskeleton and overall structure remain intact (Fig. 2a,b; Supplementary Fig. 1G; Supplementary Movies 6–10). The functional purpose for the retraction is not known, however it clearly facilitates the asymmetric inheritance of Vimentin and associated proteins during mitosis.

In order to examine the relationship between Vimentin asymmetry and the accumulation of Synphilin aggregates in differentiating mESC, we imaged live cells with RFP-tagged Vimentin and GFP-tagged Synphilin. Synphilin aggregates can be seen to colocalize with Vimentin filaments (Fig. 2c, upper panel), and remain associated with a filament as it is retracted (Fig. 2d—timelapse). In order to rule out incidental colocalization as opposed to prolonged association, we attempted to visualize colocalization between Synphilin and non-filamentous Vimentin Unit Length Filaments, and to track Synphilin aggregates on assembled Vimentin filaments over time. We employed a mutant form of Vimentin (Y117L) which is unable to assemble into filaments of cables, and instead forms Unit Length Filaments (ULFs) of 8 tetramers assembled in a barrel shape. Normally ULFs assemble end to end into a mature filament, but the mutant remains a ULF. We expressed Synphilin in Vimentin −/− cells, and co-expressed Vimentin Y117L ULFs tagged with GFP. ULFs, visualized as green puncta, associate with the surface of Synphilin aggregates, proportionally to the surface area of the aggregate (Fig. 2e, top panel for large Synphilin aggregate, lower panel for small Synphilin aggregate; quantification and statistics in Supplementary Fig. 3a).

We next visualized WT GFP-tagged Vimentin associating with RFP-tagged Synphilin aggregates prior to mitosis. Tracking a single aggregate clearly showed that Synphilin aggregates are retained by Vimentin filaments and are dragged into the Vimentin cage as Vimentin retracts to the juxtanuclear region/JUNQ inclusion (Fig. 2f, Quantification in Supplementary Fig. 3A, supplementary movie 11). Following cells through metaphase demonstrated asymmetric partitioning of the Vimentin-aggregate complex (Fig. 2f, Supplementary Movie 3 and Quantification in Fig. 2h). Vimentin −/− cells, in contrast, showed perfectly symmetrical partitioning of Synphilin aggregates (Fig. 2g, Supplementary Movie 4 and 5 and Quantification in Fig. 2h). Over time, cells lacking Vimentin accumulated significantly more Synphilin aggregates that were much larger in size (Fig. 2i). What these data cumulatively argue, is that Vimentin associates with misfolded protein aggregates, directs their accumulation in juxtanuclear Vimentin-rich structures and promotes their asymmetric partitioning during mitosis.

### Vimentin associates with misfolded proteins, stress response factors, and RNA-binding proteins during stress

The interaction between Vimentin and protein aggregates demonstrated by the above experiments prompted us to assess the Vimentin interactome systematically. For this we utilized the BioID approach^42^, consisting of fusing a promiscuous biotin Ligase, BirA* to Vimentin. This allowed us to measure the comparative interactome of Vimentin in RA differentiated cells and mESCs in distinct conditions by adding biotin following heat stress, arsenite treatment, treatment with nocodazole (to look at interaction during mitosis), as well as in control conditions.

**Figure 3:**
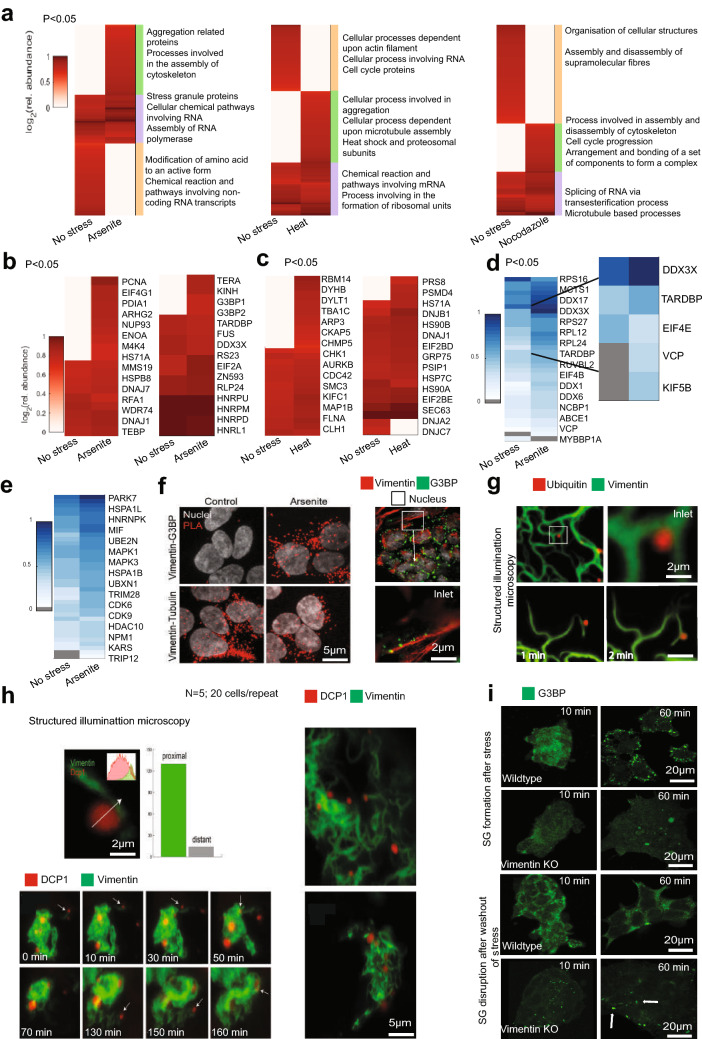
BioID of Vimentin Interactome identifies protein folding quality control components, RNA-binding proteins, and Stress Granule proteins. (**a**) Vimentin interactome in (1) No stress (2) Arsenite stress (150 μM/2 h), (3) Heat stress (44 °C/2 h) and (4) Nocodazole. GO analysis and heat maps relative to No Stress condition. (**b**) Vimentin interacts with stress response proteins (Left panel) and RNA-binding and Stress Granule proteins (Right panel) during Arsenite stress. (**c**) Vimentin interacts with proteins involved in microtubule based processes (Left panel) and heat shock, proteasomal subunits and other stress related proteins (Right panel) during heat stress. (**d**) Immunoprecipitation for Vimentin. Heat Map for RBPs binding with Vimentin. (**e**) Immunoprecipitation for Vimentin. Heat Map for stress regulating proteins binding to Vimentin. (**a**–**e**: Heat maps were plotted using established protocols in Matlab (Matlab 2019a Mathworks Ltd). https://www.mathworks.com/products/matlab.html?s_tid=hp_products_matlab. (**f**) Proximity ligation assay was performed on 4 day retinoic acid (1 μg/ml) differentiated mESCs between Vimentin—G3BP (Top panel) and Vimentin—Tubulin (positive control) (Bottom panel). PLA signal was observed between vimentin and G3BP only during arsenite stress. PLA signal was observed between vimentin and tubulin with and without arsenite stress (150 μM/2 h). (Right Panel) Confocal images of differentiated mESCs stained for G3BP (green), Vimentin (red) and Nucleus (white) during arsenite stress. The inlet image shows G3BP aggregates (green) attaching to vimentin filament (red). (**g**) Structured Illumination Microscopy images of Vimentin-GFP (green) binding to RFP ubiquitin- positive foci (red) in live cells. The interaction of Vimentin and ubiquitin persists over time shown by the time-lapse. (**h**) Vimentin-GFP (green) interacting with DCP1-RFP (a P-body component and RNA-binding protein) over time and the graph showing the association between the two. The experiment was repeated 5 times. 20 cells were selected to be imaged during each repeat. (**i**) Confocal images of Wildtype and Vimentin Knockouts differentiated with retinoic acid (1 μg/ml) fixed and stained with G3BP (green). Top panels—Stress induced with arsenite (150 μM) before fixing. Bottom Panels—Stress induced with arsenite (150 μM/1 h) and new media added to cells. The cells were fixed at 10 min and 60 min. The G3BP aggregates were observed to be dissociated after 60 min in the Wildtype cells, whereas the aggregates persisted in the Vimentin Knockout cells. **f**–**i** Images were acquired and processed using NIS elements software (version 3.2).

Comparative interactome analysis showed enrichment in specific protein networks during heat (44 °C for 2 h), arsenite stress (150 µM for 2 h), and during mitosis (nocodazole treatment for 2 h) (Fig. 3a). During stress Vimentin preferentially associated with model misfolded proteins, including Synphilin and VHL, that were ectopically expressed, confirming the cell biological evidence of interaction from Fig. 2b,d. Of the endogenous proteins that were enriched in the Vimentin interactome during stress, the two dominant networks consisted of low structural complexity RNA-binding proteins, many of which were SG components, and stress response proteins including chaperones and proteasome subunits (Fig. 3a). Interestingly, looking closely at the fold enrichment enabled a stress specific resolution of protein network enrichment. Hence, stress response proteins including chaperones and proteasome subunits were equally enriched in heat stress and during arsenite treatment (Fig. 3b), whereas Stress Granule components interacted with Vimentin more during arsenite stress than with heat stress, corresponding to conditions that promote Stress Granule formation (Fig. 3c). In addition to BioID proteomics, we confirmed the interactome with IP against Vimentin. The top overlapping hits between BioID and IP, including the Stress Granule components TARDBP, DDX3X, and VCP, are shown in Fig. 3d,e.

### Vimentin associates with the SG component G3BP, and facilitates SG formation

Confirming our interactomics we focused on aggregation-prone RNP-granule (Stress Granule and P-body) proteins and other aggregates. One of our best scoring hits during Arsenite stress was G3BP (both isoforms 1 and 2). We decided to follow up this hit because G3BP is one of the earliest Stress Granule markers. We therefore confirmed the direct interaction between G3BP with an in situ proximity ligation assay using UnFold probes^43^. The proximity ligation assay (PLA) showed association between Vimentin filaments and G3BP, but only when G3PB was assembled in Stress Granules (Fig. 3f). The Vimentin-Tubulin interaction was used as a positive control. Since tagged Ubiquitin (Ub) can be used as a proxy for following endogenous aggregate localization during heat stress, we investigated the co-localization of endogenous Vimentin (visualized with GFP-tagged chromobodies) with mCherry-tagged Ub-positive aggregates. Confocal imaging (Fig. 3g upper panels) and Structured Illumination Microscopy (SIM) (Fig. 3g, lower panels) showed proximity between Vimentin filaments and Ub-positive aggregates, which persisted over time, despite substantial cytoplasmic movement of Vimentin filaments. We also sought to confirm the interaction (detected by IP) of additional granule-forming RNA-binding proteins with Vimentin during arsenite stress. To do this, we chose a P-body component (Dcp1) and another Stress Granule component (VCP). SIM imaging once again showed direct co-localization between Vimentin filaments and P-bodies, as with Stress Granules, with nearly all P-bodies associating with a filament (Fig. 3h, upper panel). Moreover, the retraction of Vimentin filaments to the juxtanuclear space over time recruited P-bodies there as well (Fig. 3h, lower panels time-lapse; zoomed out start and finish shown in panels on the right). What’s more, Stress Granules associated with Vimentin-ULFs similarly to Synphilin aggregates (Supplementary Fig. 3B), and the complementation of a Vimentin −/− line with ULFs was sufficient to recruit Stress Granules to the juxtanuclear region (Supplementary Fig. 3B).

Extending our follow up to investigate a potential functional connection between Vimentin and RNP granule formation, we examined the effect of Vimentin KO on Stress Granule dynamics. Vimentin−/− cells showed a dramatic impairment in Stress Granule formation and clearance (Fig. 3i, quantification and statistics in Supplementary Fig. 3F), suggesting that the Vimentin-Stress Granule interaction plays a functional role in Stress Granule biology.

Since the most prominent networks of proteins enriched in the Vimentin interactome during stress were either aggregate or Stress Granule-forming proteins, or protein folding quality control proteins that associate with aggregates and granules, we wanted to verify that the interactome of a model misfolded protein overlaps with that of Vimentin during heat shock of arsenite treatment. Indeed, examining the BioID interactome of Synphilin during stress, we observed that it includes endogenous Vimentin, and overlaps strongly with the Vimentin interactome (Supplementary Fig. 3C,D,E). Together, our data argue for a stress response role for Vimentin in differentiating mESC cells.

### Vimentin is critical for mESC neuronal differentiation

**Figure 4:**
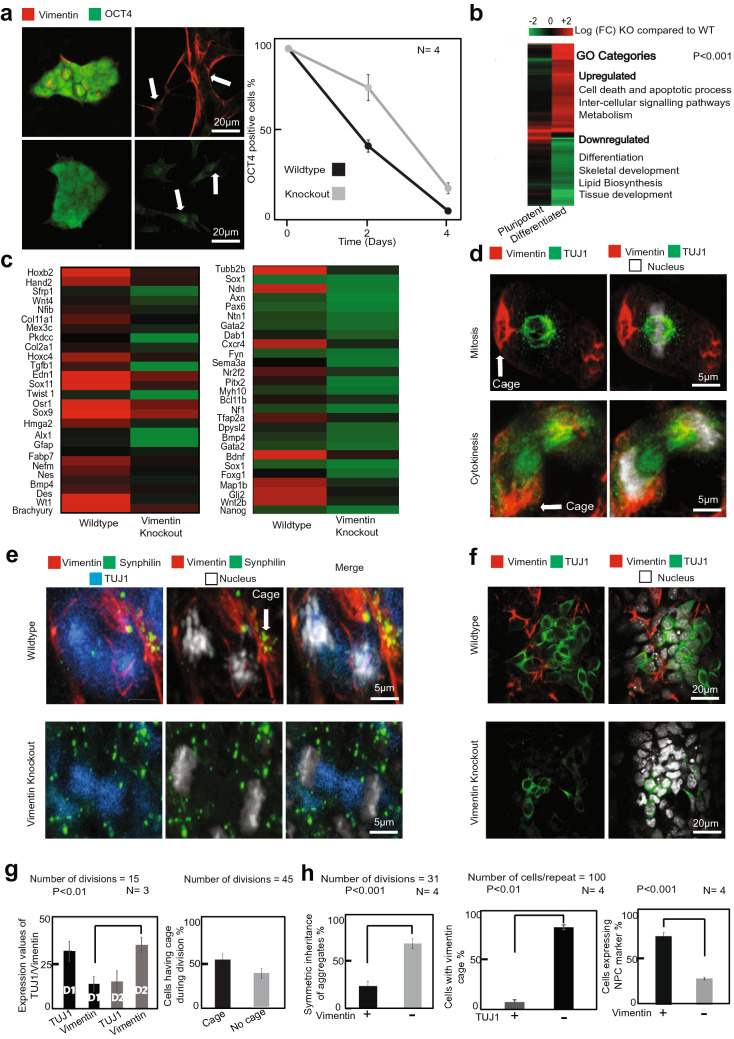
Vimentin is critical for mESC neuronal differentiation. (**a**) mESCs differentiated by the addition of retinoic acid (1 µg/ml). Number of cells expressing Oct4 after 4 days of differentiation was observed. Cells were fixed and immuno-stained for Oct4 (green) and Vimentin (red). The white arrows in the image points out that after 4 days WT cells doesn’t have OCT4 expression in the nucleus whereas vim KO cells have. The graph was plotted for percentage of cells expressing Oct4. Error bars represent standard deviation. (**b**) Whole genome RNA seq data comparison between Wildtype and Vimentin KO using Matlab software. Heat map illustrates that Vimentin Knockout does not affect pluripotent cells (first column on the left) while differentiated (by retinoic acid − 1 µg/ml) cells are affected in their gene expression patterns (right column). (**c**) Neuronal development genes downregulated in vimentin knockout cells are shown separately using a heat map. (**d**) EBs differentiated for 4 days were seeded on a different plate for neuronal progenitor cell (NPC) differentiation. Confocal images of the NPCs stained with TUJ1 (green), Vimentin (red) and nucleus (white) in vimentin wildtype cells. Statistics for (1) percentage of cells having a caged vimentin during division and (2) the expression of TUJ1 and Vimentin in the NPCs after division are shown. The error bars represent standard deviation. Statistics were done using two tailed student t-test. (**e**) Images of the misfolded protein Synphilin-GFP (green) getting asymmetrically partitioned along with Vimentin-RFP (red) in the KO complemented (top panel) and symmetrically partitioned (bottom panel) in the neuronal precursor cells. (**f**) Confocal images of NPCs stained with neural marker TUJ1 (green), Vimentin (red) and nucleus (white) in Vimentin Wildtype and Vimentin KO cells. (**g**) statistics for the expression values of vimentin and TUJ1 after division. D1 and D2 are Daughter 1 and Daughter 2 respectively. *P* < 0.01. Statistics for number of divisions with vimentin in cage/filamentous was also plotted (right panel). (**h**) Statistics for the percentage of symmetric inheritances of aggregates in Knockout complemented and Vimentin Knockouts. Statistics showing percentage of cells positive for TUJ1 marker that contain vimentin cage (middle panel) and Statistics showing percentage of cells having TUJ1 expression in wildtypes and knockouts (500 cells/replicate) and the error bars represent standard deviation (*P* < 0.01). (right panel).

Given that Vimentin has a clear role in cellular fitness, which emerges upon differentiation, we investigated the effect of Vimentin knockout on the ability of mESC to differentiate into specific lineages. Indeed, Vimentin −/− ESCs showed a slight delay in losing the OCT4 pluripotency marker (Fig. 4a), while other pluripotency genes were lost similarly to WT (Supplementary Fig. 4a). RNAseq profiling of 4-day RA-differentiated cells showed that several gene networks were up-regulated and others down-regulated in Vimentin −/− as compared to WT (Fig. 4b; Supplementary Fig. 4B). These included regulators of cell death, differentiation, metabolism, lipid synthesis, and development. However, similarly to the growth effect observed in Fig. 1, the dysregulation was only evident in RA differentiated mESC, and not in pluripotent cells (Fig. 4b).

The RNAseq profiling of RA-differentiated cells revealed that of the three germ layers, ectodermal markers were significantly lower in Vimentin KOs (Fig. 4c, Supplementary Fig. 4A). Since aggregates and misfolded proteins are thought to pose a particular danger to neurons, which arise from the ectodermal germ layer, we directly examined the ability of Vimentin −/− mESC to differentiate into Neuronal Precursor Cells (NPCs). When mESCs were differentiated along a neuronal program (Supplementary Fig. 4E), neuronal differentiation markers showed a significant difference between Vimentin KO and WT (Fig. 4c). In particular, axon extension and migration markers as well as other neuronal differentiation genes had significantly lower levels of expression in Vimentin −/− cells as compared to control (Fig. 4c).

One explanation for the neuronal differentiation defects observed in Vimentin −/− cells could be that diminished stress response impairs proper development. As cells differentiate into NPCs and neurons, they express less Vimentin, which is retained in non-differentiating cells^27^. This produces damage-free neurons and promotes survival. Indeed, Vimentin was asymmetrically partitioned in differentiated NPCs (Fig. 4d, quantification in 4g). Consequently, Vimentin −/− NPCs accumulated more Synphilin aggregates (not shown) and partitioned them symmetrically during divisions (Fig. 4e, quantification in 4h), whereas in WT cells Synphilin remained attached to Vimentin filaments and was therefore partitioned asymmetrically.

Finally, whereas WT cells immunostained for the Tuj1 (Tubb3β) neuronal lineage marker at the end of the differentiation program showed abundant expression, Vimentin −/− cells showed almost no Tuj1 expression and significantly less outgrowth of neurons from the EBs (Fig. 4f, quantification in 4h; Supplementary Fig. 4C,D). Together these data clearly argue that Vimentin is important for neuronal differentiation in mESCs. Our findings suggest that, in addition to conferring stress tolerance on differentiating cells, Vimentin may also play a signaling role in the differentiation program, apparently through its tendency to partition asymmetrically during division.

## Discussion

Cells possess the capacity to reverse the aging process that they inevitably undergo^44–46^. Cellular rejuvenation enables multi-cellular organisms to produce a pristine germline, and is also required to produce and maintain immortalized tumorigenic cell populations. Embryonic Stem Cells (ESCs) similarly have the capacity to self-renew indefinitely, and to give rise to youthful differentiated cells^47,48^. Induced pluripotency technology (iPSC) has opened a new window into cellular rejuvenation by showing that any somatic cell can be reprogrammed into an immortal pluripotent cell. Remarkably, even old cells, with aging-induced pathology, can be reprogrammed not only to become pluripotent, but to become youthful as well^49–51^.

Two mechanisms can reverse damage accumulated during aging in order to allow rejuvenation. Cells can degrade damaged components with high efficiency via the Ubiquitin–Proteasome System (UPS) and autophagy^52^. Alternatively, dividing cells can partition damaged components into one of the daughter cells during mitosis, thus generating one lineage that is pristine and one that will accumulate damage^53^. Both rejuvenation systems have been observed in developing organisms. ESCs have an elevated level of UPS function, which declines upon loss of pluripotency^54–56^. Differentiating stem cells partition misfolded and aggregated proteins away from specific lineages and into others. Neuronal precursor cells (NPCs), which divide asymmetrically into a self-renewing NPC and a differentiated neuron, partition ubiquitinated misfolded proteins away from the neurons and into the self-renewing NPC^27^.

In previous work we found that Vimentin directs the asymmetric partitioning of aggregated and ubiquitinated proteins in dividing immortal cell lines, and that this partitioning affects cell fitness^26^. It was also subsequently shown that Vimentin co-segregates with ubiquitinated protein aggregates in differentiating NPCs^27^. Another study showed that mutations in Vimentin mitotic phosphorylation sites alter neuronal differentiation in mice^57^. These findings suggest that Vimentin has an important role in cell fitness and organismal development, but mechanistic evidence for this idea has been sparse.

We examined the role of Vimentin and its ability to asymmetrically partition aggregates in the process of early differentiation of mESCs. We sought to use a system that is physiologically relevant to organismal development because previous studies have mostly not found a role of Vimentin in the organismal context, even though at the cellular level Vimentin has been implicated in many cell biological processes.

Our data show that Vimentin is, in fact, required for viability in differentiated cells (but not in stem cells); however, the requirement of Vimentin is only uncovered during stress. We further demonstrated that Vimentin protects differentiated cells from protein misfolding stress by binding aggregates and granules formed by RNA-binding, intrinsically disordered proteins, and directing their asymmetric partitioning. These results are consistent with previous findings showing that pluripotent cells have a higher level of UPS function, hence it is likely that they are able to degrade misfolded proteins more effectively without the need for asymmetric partitioning. It is not entirely clear how Vimentin interacts with aggregates and granules, but the data point to a direct interaction, as has been observed previously between Vimentin and FUS granules^58^. Since Vimentin has several disordered regions, it is possible that these directly adsorb disordered aggregates and Stress Granules^37,59^. Another interesting possibility is that Vimentin acts as a surface for nucleating Stress Granule formation. Recent reports have demonstrated a similar role for Eisosomes^60^, the Endoplasmic Reticulum^61^, and Late Endosomes^62^. The growing list of membraneless organelle-associated cellular structures opens up the fascinating possibility that different types of cytoplasmic surfaces can prime the ad hoc formation of Stress Granules where needed.

It is also possible that the extensive interactions between Vimentin and protein folding quality control machinery, including chaperones, functions to recruit aggregated proteins to Vimentin. What is clear from our data, is that Vimentin interacts with aggregates everywhere in the cytoplasm, even as a ULF, and therefore aggregates need not accumulate in the juxtanuclear region in order to be retained within the Vimentin network, as has been argued previously^34^. In fact, our data suggest that aggregates are recruited to the juxtanuclear regions as a result of their interaction with Vimentin, and not vice versa.

It is not clear whether the depletion of Synphilin aggregates in differentiating cells expressing Vimentin was solely due to asymmetric partitioning (perhaps followed by attrition of inheriting cell populations), or whether Vimentin also enhanced the degradation of aggregates. There is evidence that the accumulation of aggregates in Vimentin-associated JUNQ compartments promotes degradation^19,31^, however this is a topic for follow up study.

Our extensive comparative interactome analysis of Vimentin revealed a strikingly large number of RNA-binding proteins and Stress Granule components, as well quality control factors, interacting with Vimentin during stress. This suggests that Vimentin is an important regulator of stress response. In addition to compromising quality control, deletion of Vimentin significantly altered the differentiation program of mESCs, and severely decreased neuronal differentiation. Future experiments will reveal whether, in addition to quality control and stress response, the asymmetric partitioning of Vimentin also regulates differentiation signaling in dividing populations of stem cells.

## Materials and methods

### Cell culture

The mESC and MEF cell lines were cultured in accordance with published protocols^63^. MEFs were maintained in Dulbecco’s modified Eagle’s medium (DMEM, Sigma),10% fetal bovine serum (FBS), 2 mM L-glutamine, 50 μg/ml Penicillin and 50 μg/ml Streptomycin. R1ESCs were cultured on a feeder layer of mitomycin-C treated MEFs, and maintained in ESC culture medium (DMEM, 10% ESC-grade FBS, 2 mM L-glutamine, 1 mM sodium pyruvate, 0.1 mM nonessential amino acids, 50 μg/ml Penicillin, 50 μg/ml Streptomycin, 0.1 mM β-mercaptoethanol and 1000 U/ml (LIF). For retinoic acid (RA) induced differentiation, ESCs were grown on gelatin-coated dishes for 96 h and then plated in ESC medium without LIF supplemented with 1 µM RA. For Embryoid body differentiation (EBs) the ESCs were cultured in bacterial culture dishes in ESC media without LIF for 6 days, following the protocol previously described^63^. For neuronal precursor cells differentiation, the ESCs were plated on bacterial culture dishes without LIF for 4 days to allow for EB formation. EBs were replated on poly-L-ornithine/fibronectin (R&D Systems and Sigma respectively)-coated plates in DMEM/F12 medium (Sigma) supplemented with ITS (5 mg/ml insulin, 50 mg/ml transferrin, 30 nM selenium chloride) and fibronectin (5 mg/ml). In all differentiation protocols MEF removal was achieved by passaging the ESCs 3 times (20 min each).

### Generation of knock-out cells in mouse ES cells

Guides targeted to Vimentin gene were designed using CRISPR design tool from Zhang lab (https://crispr.mit.edu/)^64^. Appropriate target guide was selected for Vimentin gene. The sequences of guides are as follows Vimentin KO Guide FP: ACCGCCTGGTAGACATGGCTTCGA, Vimentin KO Guide RP: AACTCGAAGCCATGTCTACCAGGC. This guide was cloned into mammalian expression vector PX458; pSpCas9(BB)-2A-GFP (PX458) was a gift from Feng Zhang (Addgene plasmid # 48,138; https://n2t.net/addgene:48138; RRID:Addgene48138)^65^. Mouse R1ESCs were transfected with Vimentin expressing guide and GFP positive cells were sorted after 48 h post-transfection. The sorted cells were clonally expanded and knocking out of Vimentin gene were verified by DNA sequencing, Immuno-fluorescence, Western blotting and real-time PCR, as described earlier^64^.

### Plasmids

For transient transfection of mammalian cells, we used the plasmids DCP1-RFP (Red fluorescence protein) and UB-Cherry (ubiquitin—cherry) which drives the expression of full length CDNA of DCP1 and ubiquitin respectively. For producing the lentiviral plasmids, Vimentin–GFP (green fluorescence protein), Vimentin—RFP (red fluorescence protein), EB3—GFP (green fluorescence protein), chromobodies—RFP (red fluorescence protein), Chromobodies-GFP (green fluorescence protein)^66^, VHL-GFP, AGDD-GFP and Synphilin–GFP were subcloned into pSin-EF2-Nanog-Pur; pSin-EF2-Nanog-Pur was a gift from James Thomson (Addgene plasmid # 16,578 ; https://n2t.net/addgene:16578 ; RRID:Addgene_16578). Vimentin-BirA*-GFP and Synphilin-BirA-GFP were subcloned into pInducer-20 plasmid.

### RNA isolation, reverse transcription and quantitative real-time PCR

Total RNA from ESCs, EBs, Cardiomyocytes and RA differentiated cells was prepared as described in the RNeasy Mini Kit (Qiagen: 74,106 supplemented with RNase-free DNase set) following their instruction. Approximately 1000 ng total RNA was used for reverse transcription using the High Capacity cDNA RT kit (Applied Biosystems, 4,368,814) to make cDNA following manufacturer’s instructions with a mix of random hexamers and poly(dT) primers. Quantitative real-time PCR was performed in a BioRad sequence detection system with diluted cDNA as template. Power SYBR Green PCR Master Mix (Applied Biosystems) was used for real-time PCR.

### Genomic DNA isolation

Total genomic DNA was isolated using Sigma genomic DNA isolation kit following manufacturer’s instructions. Approximately 200 ng DNA was used for PCR to screen knock-out clones.

### Immunofluorescence (IF)

For IF experiments the cells were grown on glass coverslips in 24 well plate and fixed in 4% PFA (15 min, room temp). The fixed cells were permeabilized (0.5% Triton, 5 min, R.T.) and incubated with the primary antibodies at 4 °C over-night. Cells were then washed (X3) in PBS (5 min, R.T.), incubated with secondary antibodies (45 min at room temperature, washed again and stained with DAPI (5 min, R.T.). The coverslip was mounted in a drop of mounting solution and left for drying at room temperature and slides were stored in dark at 4 °C till imaging. The following antibodies were used (1) anti—Vimentin antibody (Santa Cruz sc-6260), (2) anti TUJ1 antibody (Santa Cruz sc-51670), (3) anti OCT4 antibody (Abcam ab-27985) and (4) G3BP (Sigma—WH0010146M1).

### RNA sequencing analysis

The RNA-seq library was made following manufacturer’s instructions. The integrity of the RNA-seq library was confirmed by running a tape-station. The analysis of RNA sequencing data was performed by mapping the raw RNA-seq reads for each condition with the reference mouse genome using TopHat programme. After running TopHat programme, the resulting alignment files were used to run Cufflinks programme that generates a transcriptome assembly file for each input condition. Subsequently, these assembled files were combined together using the Cuffmerge program to generate a merged assembly file which is used to calculate the gene and transcript expression from each sample. Finally, the reads and the merged assembly file were provided to Cuffdiff programme that calculates differential expression levels as well as tests the statistical significance.

### Affinity capture of biotinylated proteins

Cells were incubated for 24 h in complete media supplemented with 1 µg/ml doxycycline and 50 µM biotin. The cells were visualized under the microscope for Vimentin and Synphilin expression and localization. Lysis and affinity capture were performed according to^42^. After three PBS washes, cells (for small-scale analysis, < 107; for large scale analysis, 4 × 107) were lysed at 25 °C in 1 ml lysis buffer (50 mM Tris, pH 7.4, 500 mM NaCl, 0.4% SDS, 5 mM EDTA, 1 mM DTT, and 1 × Complete protease inhibitor (Roche)) and sonicated. Triton X-100 was added to 2% final concentration. After further sonication, an equal volume of 4 °C 50 mM Tris (pH 7.4) was added before additional sonication (subsequent steps at 4 °C) and centrifugation at 16,000 relative centrifugal force. 10% of the sample were saved for western blot analysis. Supernatants were incubated with 300 μl of streptavidin-coated magnetic beads (NEB). Beads were collected (subsequent steps at room temperature) and washed. Four conditions were selected to investigate the interacting proteins of Vimentin—(1) No stress (2) Arsenite stress (150 µM/2 h) (3) Heat stress (44 °C/2 h) and Nocadazole treated (10 µg/ml for 2 h). The experiments were performed in these four conditions in both pluripotent (2i media) and differentiated state (retinoic acid—1 µg/ml).

### Preparing samples for the Mass spectroscopy

The samples were prepared with minor modifications described earlier^60^.  The beads were washed twice in 25 mM Tris–HCl, pH 8.0 to remove residual detergents. Next, the beads were resuspended in 100 ul of 8 M Urea containing buffer (10 mM DTT, 25 mM Tris–HCl, pH 8.0) and incubated for 20 min, followed by addition of iodoacetamide to a concentration of 55 mM and further incubation for 20 min in the dark. Subsequently, the Urea was diluted by the addition of 6 volumes of a buffer (25 mM Tris–HCl, pH 8.0). Next, 0.25 μg trypsin was added (Promega Corp., Madison, WI, USA) to the beads and the mixture was incubated overnight at 37 °C with gentle agitation. The released peptides were desalted by loading the whole bead supernatant on C18 Stage tips. Two thirds of the eluted peptide material was used for MS analysis.

### MS data analysis

Mass spectra data were processed using the MaxQuant computational platform, version 1.5.3.12 as described earlier^60,67^. Peak lists were searched against the Mus musculus Uniprot FASTA sequence database containing a total of 9591 reviewed entries. Peptides with minimum of seven amino-acid length were considered and the required FDR was set to 1% at the peptide and protein level. Protein identification required at least 3 unique or razor peptides per protein group. Relative protein quantification in MaxQuant was performed using the label free quantification (LFQ) algorithm. LFQ in MaxQuant uses only common peptides for pair-wise ratio determination for each protein and calculates a median ratio to protect against outliers. It then determines all pair-wise protein ratios and requires a minimal number of two peptide ratios for a given protein ratio to be considered valid.

### Proximity ligation assay

PLA was performed as described previously in 4% PFA-fixed tissue or cells^43,68^. Proteins were detected by incubating the samples with specific primary and secondary antibodies conjugated with oligonucleotides after that. Ligation solution, consisting of two oligonucleotides and ligase, was added. Amplification solution, consisting of nucleotides and fluorescently labeled oligonucleotides, was added together with polymerase. Proximity ligation assays testing interactions between Vimentin and G3BP proteins (Test sample) as well as Vimentin and β-Tubulin (Control sample) were performed in mES cells according to manufacturer’s instructions using Rabbit mAB Vimentin (Cell Signaling—D21H3), Mouse mAB β-Tubulin (Cell Signaling—D3U1W) and mouse mAB G3BP (Sigma—WH0010146M1). The kit for performing the PLA was obtained from Sigma—Duolink, In Situ Red Starter Kit Mouse/Rabbit (DUO 92,101).

The interaction was quantified by measuring the expression values and plotted in the graph.

### Growth curve

Cells were grown on a 10 cm tissue culture plate and then seeded (0.1 million cells/well) to 5 cm tissue culture plate coated with gelatin. The cells were grown for 5 days. The number of cells were measured every 24 h. Cells were measured by the automatic cell counter. Graphs were plotted by using standard tools. For undifferentiated condition, the cells were grown for 4 days.

For embryoid body growth curve, the cells (1 million) were seeded on a 10 cm plate and grown for 6 days. Images were taken on the 3rd and the 6th day. Some cells were also taken and fixed on a coverslip by adding 4% para formaldehyde (20 min at 37 °C) and centrifuging them at 3000 rpm for 5 min at RT.

### Analysis of genome-wide data and proteomics data

To identify differentially expressed proteins, we compared the expression of individual proteins with the standardized (z-score) distribution of the log fold change values between each pair of samples, using all identified proteins. Statistically significant differentially expressed proteins (absolute log2 fold-change > 1; *P* < 0.05) were log2 transformed, grouped using unsupervised hierarchical clustering, and plotted using MATLAB (Matlab 2019a Mathworks Ltd) https://www.mathworks.com/products/matlab.html?s_tid=hp_products_matlab. To assess gene-set enrichment of differentially expressed proteins, we obtained a list of gene symbols for each gene ontology term from the Molecular Signatures Database (MSigDB) database. GO-annotation was done for categorizing the proteins into their respective processes they are involved in. The values were also plotted as a graph using graph pad prism software version 7 (https://www.graphpad.com/scientific-software/prism/).

### Lentiviral infection

Production of 3rd generation lentivirus was performed as described earlier^69^ using the combined ratio of transfer plasmid, packaging plasmid, Env plasmid and pRSV-Rev plasmid at 4:2:1:1, respectively. The precipitate was formed by adding 6 μg of DNA to a final volume of 500ul of non-serum media along with 20ul of PEI. The solution was briefly vortexed and incubated at room temperature for 30 min. Following this, the solution was mixed again by gentle vortexing, and then added dropwise to the cells. Flasks were rocked gently in a circular motion to distribute the precipitates, and then returned to the incubator at 5% CO2 unless otherwise stated. 10 to 12 h later, cells were gently washed once with PBS and fresh growth medium with 15% FBS were added. 24 h post-addition of the new media, the initial collection of viral supernatants was done. The conditioned medium was combined and cleared by centrifugation at 1500 rpm for 5 min at 4 °C then passed through a 0.45 μm pore PVDF Millex-HV filter. This viral supernatant media was then added to the cells needed to be infected. After infection, the cells were maintained in the viral supernatant media for 48 h and then passaged to a new plate.

### FACS cell sorting

The cells were trypsinized and new media was added to stop the reaction. The cell suspension was centrifuged at 1800 rpm for 3 min. After centrifuging the media was aspirated and PBS with 1% FBS was added to the pellet and mixed. This solution was again centrifuged again at 1800 rpm for 3 min. The supernatant was aspirated and PBS with 1% FBS was added. This tube of cell suspension was kept on ice till FACS sorting. The cell suspension was made to pass through a fluorescent activated cell sorter (FACS Aria, Becton Dickinson) to separate tagged (positive) and untagged (negative) fractions. The sorting was performed at room temperature with the laser (Coherent Innova 70) set at appropriate wavelength and 200 mW power.

### Statistical analysis

Three or more independent experiments were performed to obtain the data. Standard deviation was calculated for the population. *P* values were calculated by two-tailed Student t-test, or one-way ANOVA. The sample sizes were not predetermined. Computational and statistical analyses were performed using established protocols in Matlab (Matlab 2019a Mathworks Ltd). Imaging analysis was performed using NIS software version 3.2).

### Live cell imaging

Cells (20,000 cells) were seeded on a glass bottom 4 well plate (De Groot—76-D35C4-20). Imaging was started 24 h after the seeding of cells. Images were acquired depending upon the experiment. Cells were cultured, transfected and live cell was performed according to standard protocols^19,70^. For time-lapse imaging, we used a dual point-scanning Nikon A1R-si microscope equipped with a Piezo stage, using a 60 × PlanApo IR oil objective NA 1.4, 0.3 μm slices, and 0.2–2% laser power (from 65-mW 488-nm laser and 50-mW 561-nm laser) to acquire 3D movies. Images were acquired in resonant-scanning or Galvano-scanning mode. Each Z series was acquired with 0.5- to 1-μm step size and 10–35 steps. For super resolution Structured Illumination Microscopy (SIM) Cells were prepared as described above. Images were acquired using a Nikon nSIM microscope in 2D mode with a 488 nm and 561 nm lasers. A 100 × oil TIRF objective (NA 1.49) was used for the imaging. Prior to imaging the point-spread function was visualized with 100 nm fluorescence beads in order to adjust the correction ring of the objective to the coverslip thickness. The final image was reconstructed using NIS-Elements software (Version 4.1).

## Supplementary information

Supplementary Legends.

Supplementary Figures.

Supplementary Video 1.

Supplementary Video 2.

Supplementary Video 3.

Supplementary Video 4.

Supplementary Video 5.

Supplementary Video 6.

Supplementary Video 7.

Supplementary Video 8.

Supplementary Video 9.

Supplementary Video 10.

Supplementary Video 11.

## References

[1] Castro-Muñozledo F, Meza-Aguilar DG, Domínguez-Castillo R, Hernández-Zequinely V, Sánchez-Guzmán E (2017). Vimentin as a marker of early differentiating, highly motile corneal epithelial cells. J. Cell. Physiol..

[2] Werner WF, Christine G, Caecilia K, Brian WJ, Karl I (1982). Formation of cytoskeletal elements during mouse embryogenesis. Differentiation.

[3] Qin Z, Buehler MJ (2011). Structure and dynamics of human vimentin intermediate filament dimer and tetramer in explicit and implicit solvent models. J. Mol. Model..

[4] Strelkov SV, Herrmann H, Aebi U (2003). Molecular architecture of intermediate filaments. BioEssays.

[5] Gan Z, Ding L, Burckhardt CJ (2016). Vimentin Intermediate Filaments Template Microtubule Networks to Enhance Persistence in Cell Polarity and Directed Migration. Cell Syst..

[6] Matveeva EA, Venkova LS, Chernoivanenko IS, Minin AA (2015). Vimentin is involved in regulation of mitochondrial motility and membrane potential by Rac1. Biol. Open.

[7] Heid H, Rickelt S, Zimbelmann R, Winter S, Schumacher H (2014). On the formation of lipid droplets in human adipocytes: the organization of the perilipin-vimentin cortex. PLoS ONE.

[8] Nicholl ID, Quinlan RA (1994). Chaperone activity of α-crystallins modulates intermediate filament assembly. EMBO J..

[9] Toivola DM, Strnad P, Habtezion A, Omary MB (2010). Intermediate filaments take the heat as stress proteins. Trends Cell Biol..

[10] Perlson E (2006). Vimentin binding to phosphorylated Erk sterically hinders enzymatic dephosphorylation of the kinase. J. Mol. Biol..

[11] Pérez-Sala D, Oeste CL, Martínez AE, Carrasco MJ, Garzón B, Cañada FJ (2015). Vimentin filament organization and stress sensing depend on its single cysteine residue and zinc binding. Nat Commun..

[12] Guo M (2013). The role of vimentin intermediate filaments in cortical and cytoplasmic mechanics. Biophys J..

[13] Eckes B (1998). Impaired mechanical stability, migration and contractile capacity in vimentin-deficient fibroblasts. J. Cell Sci..

[14] Eckes B (2000). Impaired wound healing in embryonic and adult mice lacking vimentin. J. Cell Sci..

[15] Terzi F (1997). Reduction of renal mass is lethal in mice lacking vimentin Role of endothelin-nitric oxide imbalance. J. Clin. Invest..

[16] Moisan E, Chiasson S, Girard D (2007). The intriguing normal acute inflammatory response in mice lacking vimentin. Clin. Exp. Immunol..

[17] Boraas LC, Ahsan T (2016). Lack of vimentin impairs endothelial differentiation of embryonic stem cells. Sci. Rep..

[18] Tolstonog GV, Shoeman RL, Traub U, Traub P (2001). Role of the intermediate filament protein vimentin in delaying senescence and in the spontaneous immortalization of mouse embryo fibroblasts. DNA Cell Biol..

[19] Mendez MG, Kojima S, Goldman RD (2010). Vimentin induces changes in cell shape, motility, and adhesion during the epithelial to mesenchymal transition. FASEB J..

[20] Kokkinos MI (2007). Vimentin and epithelial-mesenchymal transition in human breast cancer–observations in vitro and in vivo. Cells Tissues Organs..

[21] Jiu Y (2017). Vimentin intermediate filaments control actin stress fiber assembly through GEF-H1 and RhoA. J. Cell Sci..

[22] Danielsson F, Peterson MK, Caldeira Araujo H, Lautenschlager F, Gad AKB (2018). Vimentin diversity in health and disease. Cells.

[23] Antfolk D (2017). Selective regulation of Notch ligands during angiogenesis is mediated by vimentin. Proc. Natl. Acad. Sci. USA.

[24] Haversen L (2018). Vimentin deficiency in macrophages induces increased oxidative stress and vascular inflammation but attenuates atherosclerosis in mice. Sci. Rep..

[25] Hol EM, Capetanaki Y (2017). Type III Intermediate Filaments Desmin, Glial Fibrillary Acidic Protein (GFAP), Vimentin, and Peripherin. Cold Spring Harb. Perspect. Biol..

[26] Ogrodnik M (2014). Dynamic JUNQ inclusion bodies are asymmetrically inherited in mammalian cell lines through the asymmetric partitioning of vimentin. Proc. Natl. Acad. Sci. USA.

[27] Moore DL, Pilz GA, Arauzo-Bravo MJ, Barral Y, Jessberger S (2015). A mechanism for the segregation of age in mammalian neural stem cells. Science.

[28] Spokoini R (2012). Confinement to organelle-associated inclusion structures mediates asymmetric inheritance of aggregated protein in budding yeast. Cell Rep..

[29] Shcheprova Z, Baldi S, Frei SB, Gonnet G, Barral Y (2008). A mechanism for asymmetric segregation of age during yeast budding. Nature.

[30] Pattabiraman S, Kaganovich D (2014). Imperfect asymmetry: The mechanism governing asymmetric partitioning of damaged cellular components during mitosis. Bioarchitecture.

[31] Erjavec N, Cvijovic M, Klipp E, Nystrom T (2008). Selective benefits of damage partitioning in unicellular systems and its effects on aging. Proc. Natl. Acad. Sci. U S A.

[32] Amen T, Kaganovich D (2015). Dynamic droplets: the role of cytoplasmic inclusions in stress, function, and disease. Cell. Mol. Life Sci..

[33] Cogne, B. et al*.* A dominant vimentin variant causes a rare syndrome with premature aging. *European journal of human genetics : EJHG* (2020) 10.1038/s41431-020-0583-2 PMC7609319 32066935

[34] Johnston JA, Ward CL, Kopito RR (1998). Aggresomes: a cellular response to misfolded proteins. J Cell Biol..

[35] Kaganovich D (2017). There is an inclusion for that: material properties of protein granules provide a platform for building diverse cellular functions. Trends Biochem. Sci..

[36] Weisberg SJ (2012). Compartmentalization of superoxide dismutase 1 (SOD1G93A) aggregates determines their toxicity. Proc. Natl. Acad. Sci. U.S.A..

[37] Miyazaki Y, Mizumoto K, Dey G (2016). A method to rapidly create protein aggregates in living cells. Nat. Commun..

[38] Zaarur N, Meriin AB, Gabai VL, Sherman MY (2008). Triggering Aggresome formation. J. Biol. Chem..

[39] Mathew A, Mathur SK, Jolly C, Fox SG, Kim S, Morimoto RI (2001). Stress-specific activation and repression of heat shock factors 1 and 2. Mol. Cell Biol..

[40] Rathje LS (2014). Oncogenes induce a vimentin filament collapse mediated by HDAC6 that is linked to cell stiffness. Proc. Natl. Acad. Sci. USA.

[41] Jiu Y (2015). Bidirectional interplay between vimentin intermediate filaments and contractile actin stress fibers. Cell Rep..

[42] Roux KJ, Kim DI, Raida M, Burke B (2012). A promiscuous biotin ligase fusion protein identifies proximal and interacting proteins in mammalian cells. J. Cell Biol..

[43] Klaesson A (2018). Improved efficiency of in situ protein analysis by proximity ligation using UnFold probes. Sci. Rep..

[44] Rujano MA (2006). Polarised asymmetric inheritance of accumulated protein damage in higher eukaryotes. PLoS Biol..

[45] Erjavec N, Nystrom T (2007). Sir2p-dependent protein segregation gives rise to a superior reactive oxygen species management in the progeny of Saccharomyces cerevisiae. Proc. Natl. Acad. Sci. USA.

[46] Coelho M, Lade SJ, Alberti S, Gross T, Tolic IM (2014). Fusion of protein aggregates facilitates asymmetric damage segregation. PLoS Biol..

[47] Yamashita YM, Fuller MT, Jones DL (2005). Signaling in stem cell niches: lessons from the Drosophila germline. J. Cell Sci..

[48] Clevers H (2005). Stem cells, asymmetric division and cancer. Nat. Genet..

[49] Avior Y, Sagi I, Benvenisty N (2016). Pluripotent stem cells in disease modelling and drug discovery. Nat. Rev. Mol. Cell Biol..

[50] Leor J (2007). Human embryonic stem cell transplantation to repair the infarcted myocardium. Heart.

[51] Takahashi K (2007). Induction of pluripotent stem cells from adult human fibroblasts by defined factors. Cell.

[52] Lilienbaum A (2013). Relationship between the proteasomal system and autophagy. Int. J. Biochem. Mol. Biol..

[53] Bufalino MR, van der Kooy D (2014). The aggregation and inheritance of damaged proteins determines cell fate during mitosis. Cell Cycle.

[54] Vilchez D (2012). Increased proteasome activity in human embryonic stem cells is regulated by PSMD11. Nature.

[55] Selenina AV, Tsimokha AS, Tomilin AN (2017). Proteasomes in Protein Homeostasis of Pluripotent Stem Cells. Acta Nat..

[56] Saez I, Koyuncu S, Gutierrez-Garcia R, Dieterich C, Vilchez D (2018). Insights into the ubiquitin-proteasome system of human embryonic stem cells. Sci. Rep..

[57] Chen M (2018). Increased neuronal differentiation of neural progenitor cells derived from phosphovimentin-deficient mice. Mol. Neurobiol..

[58] Lin Y (2016). Toxic PR poly-dipeptides encoded by the C9orf72 repeat expansion target LC domain polymers. Cell.

[59] England JL, Kaganovich D (2011). Polyglutamine shows a urea-like affinity for unfolded cytosolic protein. FEBS Lett..

[60] Amen T, Kaganovich D (2020). Stress granules sense metabolic stress at the plasma membrane and potentiate recovery by storing active Pkc1. Sci. Signal..

[61] Lee JE, Cathey PI, Wu H, Parker R, Voeltz GK (2020). Endoplasmic reticulum contact sites regulate the dynamics of membraneless organelles. Science.

[62] Cioni JM, Lin JQ, Holtermann AV (2019). Late endosomes act as mRNA translation platforms and sustain mitochondria in axons. Cell.

[63] Alajem A (2015). Differential association of chromatin proteins identifies BAF60a/SMARCD1 as a regulator of embryonic stem cell differentiation. Cell Rep.

[64] Azad GK (2018). PARP1-dependent eviction of the linker histone H1 mediates immediate early gene expression during neuronal activation. J. Cell Biol..

[65] Ran F, Hsu P, Wright J (2013). Genome engineering using the CRISPR-Cas9 system. Nat Protoc..

[66] Maier J, Traenkle B, Rothbauer U (2015). Real-time analysis of epithelial-mesenchymal transition using fluorescent single-domain antibodies. Sci. Rep..

[67] Tyanova S, Temu T, Cox J (2016). The MaxQuant computational platform for mass spectrometry-based shotgun proteomics. Nat. Protoc..

[68] Zhang W, Xie M, Shu MD, Steitz JA, DiMaio D (2016). A proximity-dependent assay for specific RNA-protein interactions in intact cells. RNA.

[69] Cribbs AP, Kennedy A, Gregory B, Brennan FM (2013). Simplified production and concentration of lentiviral vectors to achieve high transduction in primary human T cells. BMC Biotechnol..

[70] Oeser ML (2016). Dynamic sumoylation of a conserved transcription corepressor prevents persistent inclusion formation during hyperosmotic stress. PLoS Genet..

