# Supplementary material for: Vimentin protects differentiating stem cells from stress
Source: Sci Rep. 2020 Nov 11;10:19525. doi: 10.1038/s41598-020-76076-4 (PMC7658978; doi:10.1038/s41598-020-76076-4)
Supplement: Supplementary file 1 — Supplementary Legends. [file 41598_2020_76076_MOESM1_ESM.docx]

**Supplementary Figure 1. CRISPR-CAS9 KO of Vimentin** (A) Expression of Vimentin increases during differentiation (quantification of Vimentin IF). Error bars represent standard deviation. (B) Raw western blot images for the Vimentin knockout confirmation and the vimentin protein enrichment during differentiation. The corresponding statistics is also shown. (C) Confirmation of genomic DNA sequencing. Primers were designed and the extracted genomic DNA was sequenced for the three selected clones. (D) Confirmation by Immuno-fluorescence. (E) Confirmation by RT PCR. Four different primers were designed and checked on the clones of knockouts. (F) Raw western blot images for the HSF-1 protein enrichment during in wildtype and vimentin knockout cell lines during different conditions. (G) Cells lentivirally infected with anti- Vimentin chromobodies (RFP), EB3 (GFP) and Nucleus (IRFP, shown in blue). Arsenite stress (150µm/2 hours) was induced before fixing. Images were acquired and processed using NIS elements software (version 3.2)

**Supplementary Figure 2. Defective EB growth and poor stress tolerance in Vimentin KO cells.** (A and B) images showing the EB growth for Wildtype, KO, and KO complements with and without ectopic expression of synphilin, with and without heat stress.

**Supplementary Figure 3. Vimentin and Synphilin interactomes.** (A) Statistics comparing (1) colonies with aggregates (100 colonies were observed during each replicate) and (2) size of the aggregates, between the cells with vimentin and without vimentin (100 cells with aggregates were taken and the aggregate sizes were measured). Error bars represent standard deviation. Statistics were done by two tailed t-test. (A) Confocal images of vimentin–ULF-GFP (green) interacting with VCP-RFP (endogenously tagged, red) during arsenite stress (100μM, 10 minutes). (B) Images of VCP (red) colocalizing with Vimentin–ULF (green) in the juxtanuclear region. (C, D and E) Vimentin and Synphilin bind to the same set of stress response, ribosomal, and RNA-binding proteins. Graphs plotted using graph pad prism version 7 (https://www.graphpad.com/scientific-software/prism/).

(F) Statistics comparing the rate of formation of stress granule aggregates (G3BP) after stress and stress washout in Wildtype and Vimentin Knockout cells were plotted.
Images of colonies of wildtype and knockout cells before the addition of stress (0min) data are shown. Images were acquired and processed using NIS elements software (version 3.2)

**Supplementary Figure 4. Vimentin is required for neuronal differentiation.** (A) RT PCR of genes of all the three germ layers. (B) Brightfield images of defective NPC outgrowth in Vimentin KO cells compared to Wildtype and KO complemented cells after 3 days of NPC differentiation. (C) Quantification showing (1) number of EBs getting attached after culturing in NPC media (2) the size of the NPC projection coming out of the EBs after getting attached to the plate. Error bars represent standard deviation. (D) Schematic of NPC differentiation protocol. Statistics were done using two tailed student t-test.

**Supplementary movie 1:** Cells were lentivirally infected to express H2B – IRFP (Shown in green) and anti-vimentin chromobody (RFP). The cells undergo division where vimentin in filamentous form is asymmetrically during mitosis. The frames were imaged at 10min apart.

**Supplementary movie 2:** Cells were lentivirally infected to express H2B – IRFP (Shown in white) and anti-vimentin chromobody (RFP). The cells (C1) undergo multiple division. We can observe from the timelapse that, the vimentin collapses before each division to be asymmetrically inherited. The daughter cell (D1), which inherited less vimentin, begins to form the vimentin in its juxtanuclear position before mitosis ultimately to asymmetric vimentin inheritance during mitosis (D21/D12). The frames were imaged at 10min apart

**Supplementary movie 3:** Cells were lentivirally infected to express anti-vimentin chromobody (RFP) and Synphilin (GFP). Vimentin mediates the asymmetric inheritance of the synphilin aggregates. The frames were imaged at 10min apart

**Supplementary movie 4:** Vimentin knockout cells were lentivirally infected to express anti-vimentin chromobody (RFP), H2B – IRFP (shown in white) and Synphilin (GFP). Aggregates are symmetrically inherited during mitosis. The frames were imaged at 10min apart

**Suppementary movie 5:** Vimentin knockout cells were lentivirally infected to express anti-vimentin chromobody (RFP) and VHL (GFP). Aggregates are symmetrically inherited during mitosis. The frames were imaged at 10min apart

**Supplementary movie 6:** Cells were lentivirally infected to express EB3 (GFP) and anti-vimentin chromobody (RFP). The cells were induced with arsenite stress (150μM) prior to the start of imaging. The frames were imaged at 10min apart

**Supplementary movie 7:** Vimentin wildtype cells were lentivirally infected to express anti-vimentin chromobody (RFP) and synphilin (GFP). The cells were induced with arsenite stress (150μM) prior to the start of imaging. We could observe that, vimentin collapses along with synphilin aggregated proteins bringing them to the vimentin cage in the juxta-nuclear position. The frames were imaged at 10min apart.

**Supplementary movie 8:** Vimentin wildtype cells were lentivirally infected to express anti-vimentin chromobody (RFP) and VHL (GFP). The cells were induced with arsenite stress (150μM) prior to the start of imaging. We could observe that, vimentin collapses along with VHL aggregated proteins bringing them to the vimentin cage in the juxta-nuclear position. The frames were imaged at 10min apart.

**Supplementary movie 9:** Cells were lentivirally infected to express anti-vimentin chromobody (RFP), H2B – IRFP (shown in white) and AGDD (GFP). We could observe that, vimentin collapses along with AGDD aggregated proteins bringing them to the vimentin cage in the juxta-nuclear position. The frames were imaged at 10min apart.

**Supplementary movie 10:** Cells were lentivirally infected to express anti-vimentin chromobody (RFP), H2B – IRFP (shown in blue) and EB3(GFP). We could observe that, vimentin in the collapsed form is asymmetrically inherited to one of the daughter cells. The frames were imaged at 10min apart.

**Supplementary movie 11:** Cells were lentivirally infected to express anti-vimentin chromobody (GFP) and Synphilin(RFP). It is observed that, through time, synphilin is attached to the vimentin filament and is recruited to the vimentin cage. The frames for the movie was continuously acquired to observe the movement of the aggregates along with the vimentin filament.
