# Supplementary material for: Vimentin protects differentiating stem cells from stress
Source: Sci Rep. 2020 Nov 11;10:19525. doi: 10.1038/s41598-020-76076-4 (PMC7658978; doi:10.1038/s41598-020-76076-4)
Supplement: Supplementary file 2 — Supplementary Figures. [file 41598_2020_76076_MOESM2_ESM.pdf]

Supplementary material

Vimentin protects differentiating stem cells from stress

Sundararaghavan Pattabiraman[1], Gajendra Kumar Azad[2,4], Triana Amen[1], Shlomi Brielle[1], Jung Eun Park[5], Siu Kwan Sze[5], Eran Meshorer[2,3], and Daniel Kaganovich[1]

1. Department of Experimental Neurodegeneration, University Medical Center Göttingen, Waldweg 33, 37073 Göttingen, Germany

2. Department of Genetics, The Institute of Life Sciences, The Hebrew University of Jerusalem, Edmond J. Safra Campus, Jerusalem 91904, Israel

3. The Edmond and Lily Safra Center for Brain Research (ELSC), The Hebrew University of Jerusalem, Edmond J. Safra Campus, Jerusalem 91904, Israel

4. Current address: Department of Zoology, Patna University, Patna 800005, India

5. School of Biological Sciences, Nanyang Technological University, 60 Nanyang Drive, Singapore 637551, Singapore

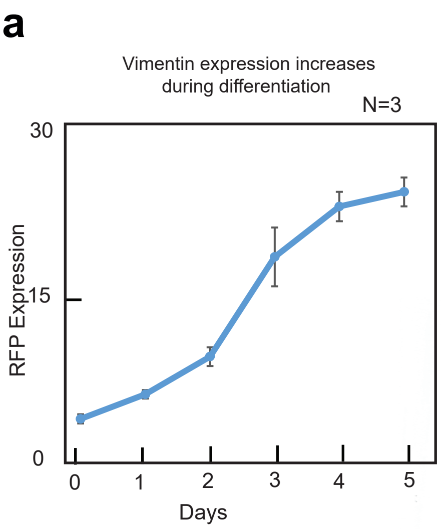

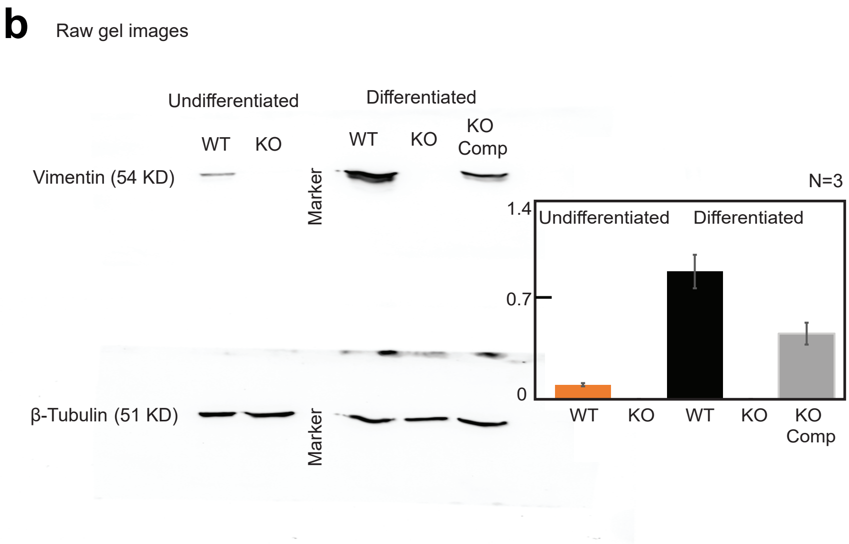

c Confirmation by genomic DNA sequencing

WT
:AGTCCTCTGCCACTCTTGCTCCGGGACCCCAGAGACCCCAGCGCTCCTACGATTCACAGCCACCGCGCCCTCATTCCCTTGTTGCAGTTTTTCCA
GCCGCAGCAAGCCAGCCCACCTTCGAAGCCATGTCTACCAGGTCTGTGTCCTCGTCCTCCTACCGCAGGATGTTCGGTGGCTCCGGCACATCGA
GCCGGCCCAGCTCCAACCGGAGCTATGTGACCACGTCCACACGCACCTACAGTCTGGGCAGCGCACTGCGCCCCAGCACTAGCCGCAGCCTCT
ATTCCTCATCCCCCGGTGGCGCCTATGTGACCCGGTCCTCGGCAGTGCGCCTGCGGAGCAGCGTGCCGGGCGTGCGGCTGCTTCAAGACTCGG
TGGACTTCTCGCTGGCCGACGCC

KO5
:AGTCCTCTGCCACTCTTGCTCCGGGACCCCAGAGACCCCAGCGCTCCTACGATTCACAGCCACCGCGCCCTCATTCCCTTGTTGCAGTTTTTCCA
GCCGCAGCAAGCCAGCCCACCTTC-------------264 nt deleted---
---TCGCTGGCCGACGCC

KO13:AGTCCTCTGCCTC--- --
--315 nt deleted---GTGCGCCT
GCGGAGCAGCGTGCCGGGCGTGCGGCTGCTTCAAGACTCGGTGGACTTCTCGCTGGCCGACGCC

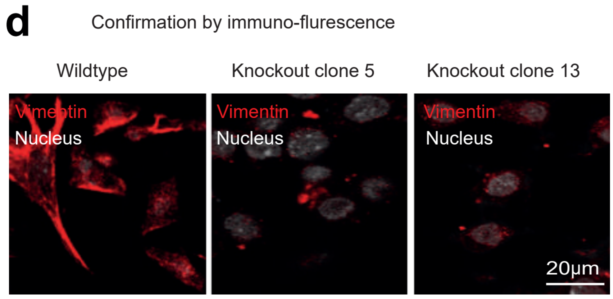

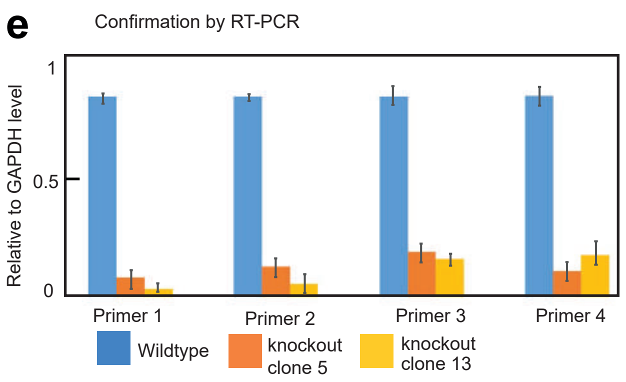

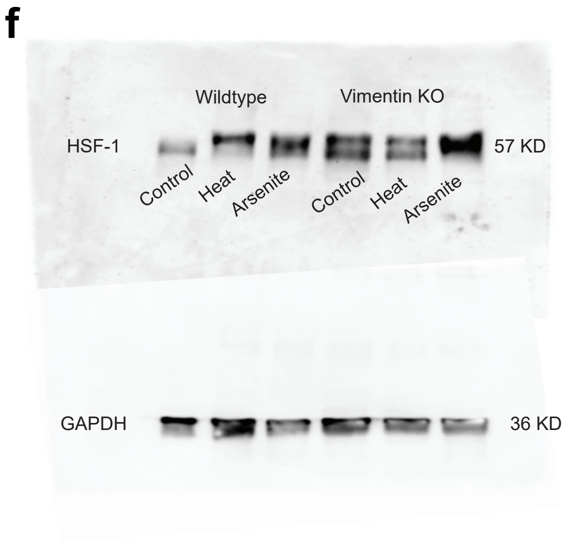

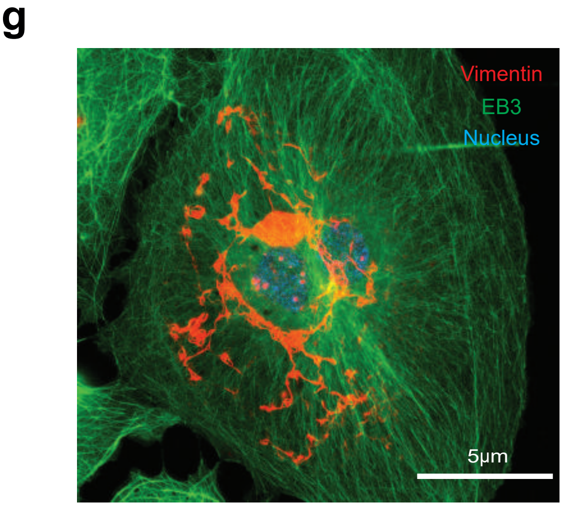

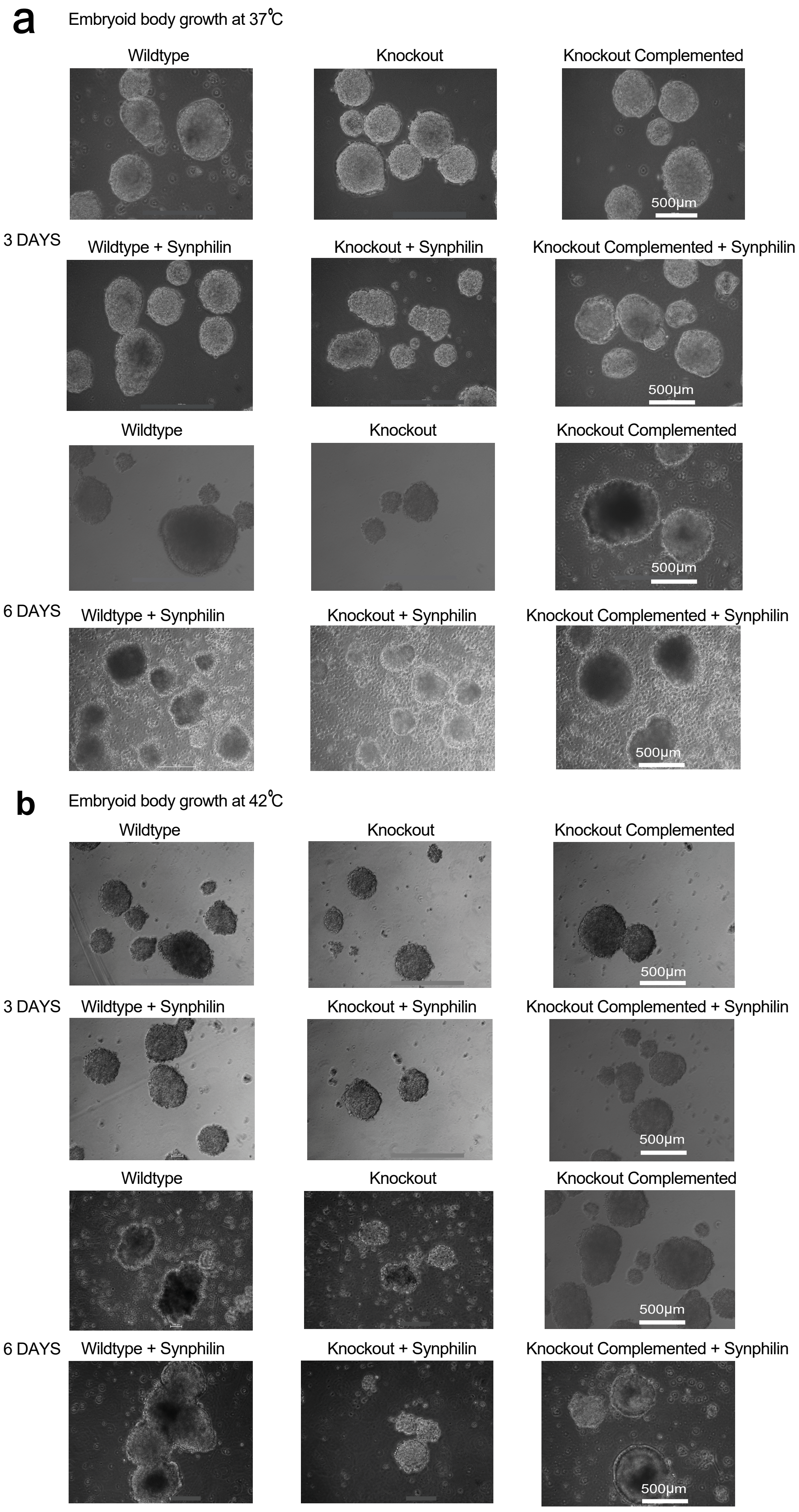
a
Embryoid body growth at 37°C
Wildtype
Knockout
Knockout Complemented
3 DAYS
Wildtype + Synphilin
Knockout + Synphilin
Knockout Complemented + Synphilin
Wildtype
Knockout
Knockout Complemented
6 DAYS
Wildtype + Synphilin
Knockout + Synphilin
Knockout Complemented + Synphilin
500μm
b
Embryoid body growth at 42°C
Wildtype
Knockout
Knockout Complemented
3 DAYS
Wildtype + Synphilin
Knockout + Synphilin
Knockout Complemented + Synphilin
Wildtype
Knockout
Knockout Complemented
6 DAYS
Wildtype + Synphilin
Knockout + Synphilin
Knockout Complemented + Synphilin
500μm

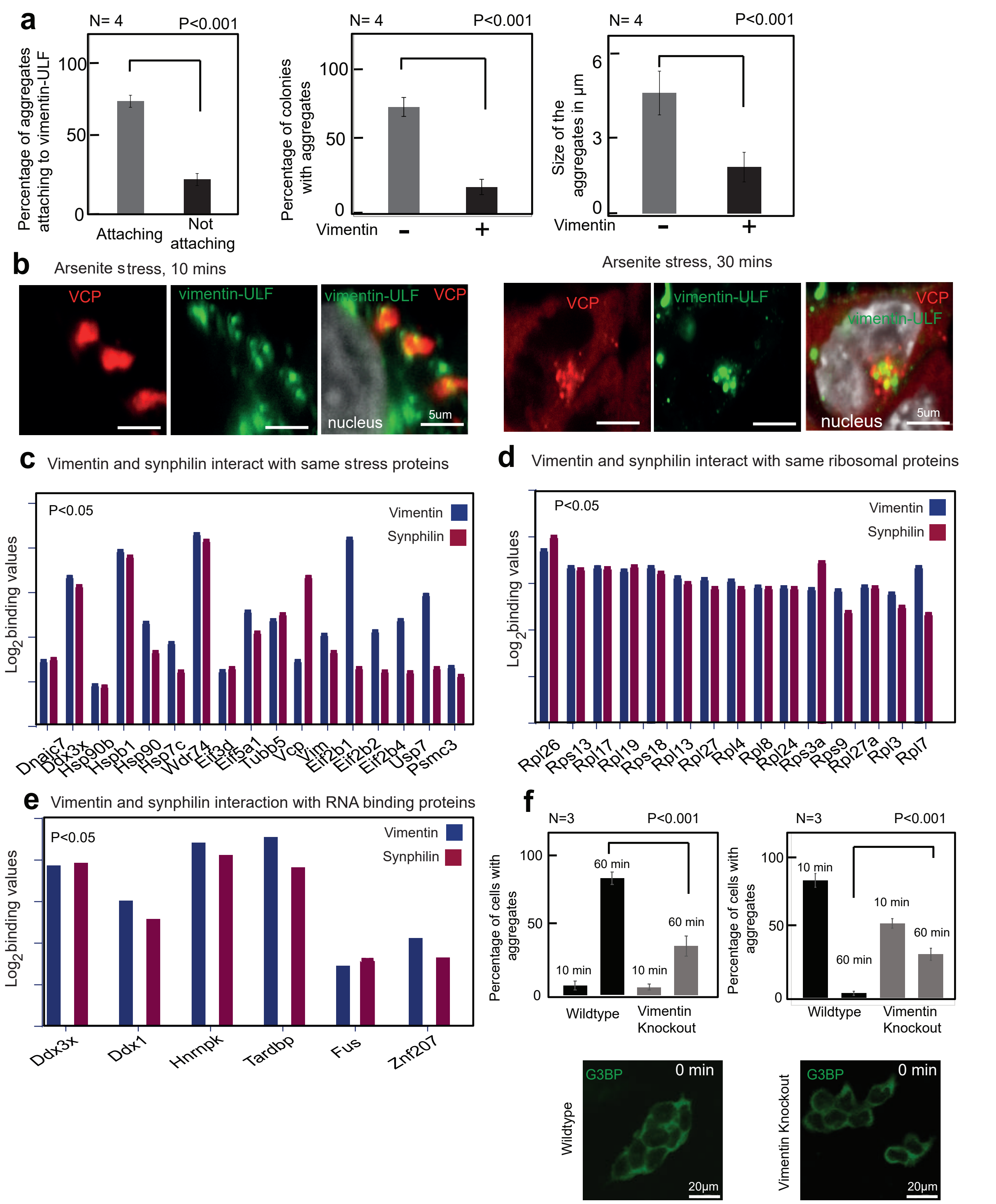
a
N= 4
P<0.001
Percentage of aggregates attaching to vimentin-ULF
100
50
0
Attaching
Not attaching
Percentage of colonies with aggregates
Vimentin
-
+
Size of the aggregates in µm
6
3
b
Arsenite stress, 10 mins
Arsenite stress, 30 mins
VCP
vimentin-ULF
nucleus
5um
c
Vimentin and synphilin interact with same stress proteins
P<0.05
Vimentin
Synphilin
Log2binding values
Dnajc7
Ddx3x
Hsp90b
Hspb1
Hsp90
Hsp7c
Wdr74
Eif3d
Eif5a1
Tubb5
Vcp
Vim
Eif2b1
Eif2b2
Eif2b4
Usp7
Psmc3
d
Vimentin and synphilin interact with same ribosomal proteins
Rpl26
Rps13
Rpl17
Rpl19
Rps18
Rpl13
Rpl27
Rpl4
Rpl8
Rpl24
Rps3a
Rps9
Rpl27a
Rpl3
Rpl7
e
Vimentin and synphilin interaction with RNA binding proteins
Ddx3x
Ddx1
Hnrnpk
Tardbp
Fus
Znf207
f
N=3
Percentage of cells with aggregates
10 min
60 min
Wildtype
Vimentin Knockout
G3BP
0 min
20µm

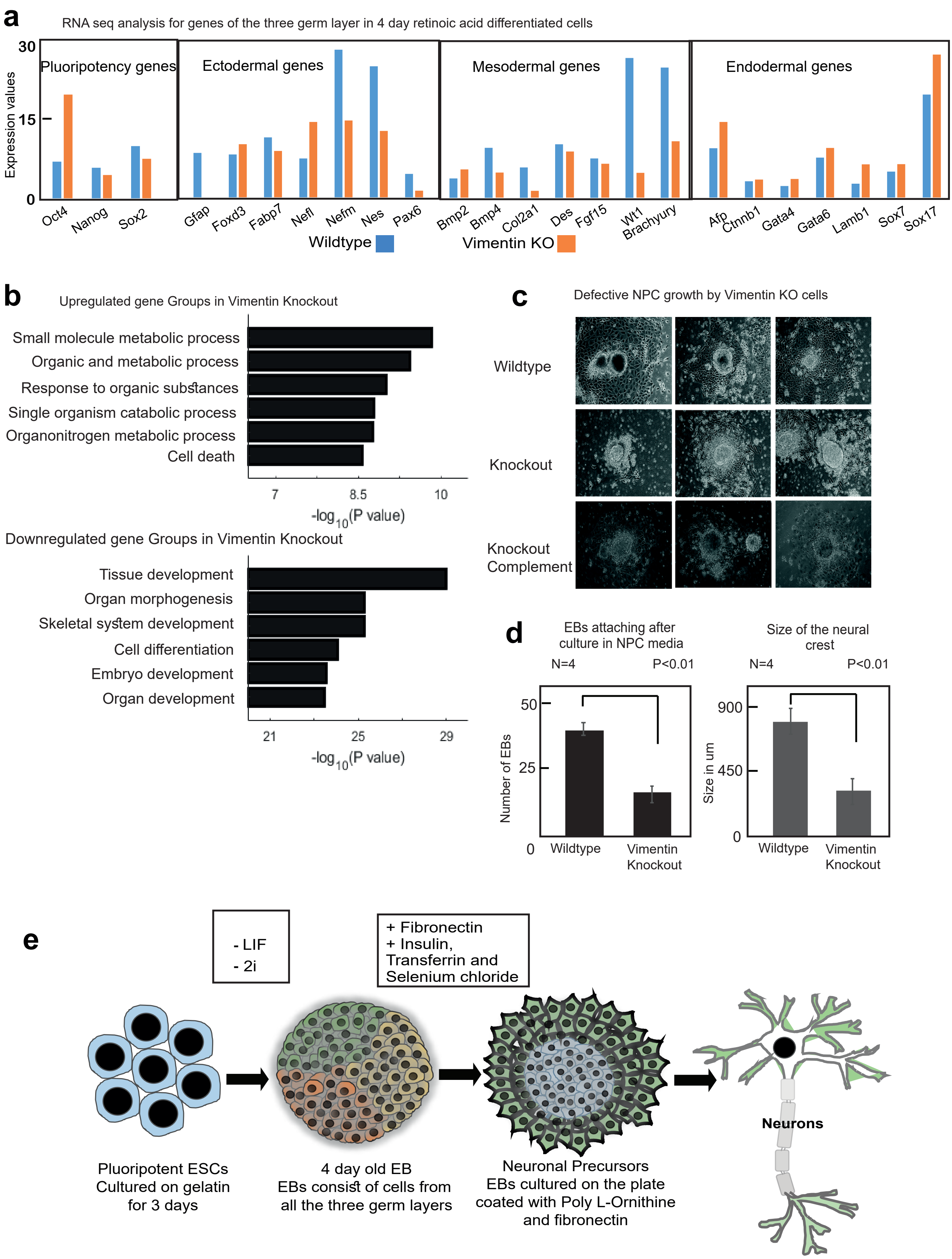
a
RNA seq analysis for genes of the three germ layer in 4 day retinoic acid differentiated cells
Expression values
30
15
0
Pluripotency genes
Ectodermal genes
Mesodermal genes
Endodermal genes
Oct4
Nanog
Sox2
Gfap
Foxd3
Fabp7
Nefl
Nefm
Nes
Pax6
Bmp2
Bmp4
Col2a1
Des
Fgf15
Wt1
Brachyury
Afp
Ctnnb1
Gata4
Gata6
Lamb1
Sox7
Sox17
Wildtype
Vimentin KO
b
Upregulated gene Groups in Vimentin Knockout
Small molecule metabolic process
Organic and metabolic process
Response to organic substances
Single organism catabolic process
Organonitrogen metabolic process
Cell death
7
8.5
10
-log10(P value)
Downregulated gene Groups in Vimentin Knockout
Tissue development
Organ morphogenesis
Skeletal system development
Cell differentiation
Embryo development
Organ development
21
25
29
-log10(P value)
c
Defective NPC growth by Vimentin KO cells
Wildtype
Knockout
Knockout Complement
d
EBs attaching after culture in NPC media
N=4
P<0.01
Number of EBs
50
25
0
Wildtype
Vimentin Knockout
Size of the neural crest
N=4
P<0.01
Size in um
900
450
0
Wildtype
Vimentin Knockout
e
- LIF
- 2i
+ Fibronectin
+ Insulin,
Transferrin and
Selenium chloride
Pluoripotent ESCs
Cultured on gelatin
for 3 days
4 day old EB
EBs consist of cells from
all the three germ layers
Neuronal Precursors
EBs cultured on the plate
coated with Poly L-Ornithine
and fibronectin
Neurons
